# The Use of Crisis Services Following the Mass School Shooting in Uvalde, Texas: Quasi-Experimental Event Study

**DOI:** 10.2196/42811

**Published:** 2023-02-08

**Authors:** Kirsty J Weitzel, Robert F Chew, Adam Bryant Miller, Caroline W Oppenheimer, Ashley Lowe, Anna Yaros

**Affiliations:** 1 Center for Data Science and AI RTI International Research Triangle Park, NC United States; 2 Center on Social Determinants, Risk Behaviors & Prevention Science RTI International Research Triangle Park, NC United States; 3 Department of Psychology & Neuroscience University of North Carolina at Chapel Hill Chapel Hill, NC United States; 4 Transformative Research Unit for Equity RTI International Research Triangle Park, NC United States

**Keywords:** mass shooting, Crisis Text Line, firearms, mental health, suicide, trauma, indirect exposure, public health, crisis management, mental health support, mass casualty, shooting, crisis service

## Abstract

**Background:**

Mass shootings result in widespread psychological trauma for survivors and members of the affected community. However, less is known about the broader effects of indirect exposure (eg, media) to mass shootings. Crisis lines offer a unique opportunity to examine real-time data on the widespread psychological effects of mass shootings.

**Objective:**

Crisis Text Line is a not-for-profit company that provides 24/7 confidential SMS text message–based mental health support and crisis intervention service. This study examines changes in the volume and composition of firearm-related conversations at Crisis Text Line before and after the mass school shooting at Robb Elementary School on May 24, 2022, in Uvalde, Texas.

**Methods:**

A quasi-experimental event study design was used to compare the actual volume of firearm-related conversations received by Crisis Text Line post shooting to forecasted firearm conversation volume under the counterfactual scenario that a shooting had not occurred. Conversations related to firearms were identified among all conversations using keyword searches. Firearm conversation volume was predicted using a seasonal autoregressive integrated moving average model trained on the 3 months of data leading up to the shooting. Additionally, proportions of issue tags (topics coded post conversation by volunteer crisis counselors at Crisis Text Line after the exchange) were compared in the 4 days before (n=251) and after (n=417) the shooting to assess changes in conversation characteristics. The 4-day window was chosen to reflect the number of days conversation volume remained above forecasted levels.

**Results:**

There was a significant increase in the number of conversations mentioning firearms following the shooting, with the largest spike (compared to forecasted numbers) occurring the day after the shooting (n=159) on May 25, 2022. By May 28, the volume reverted to within the 95% CI of the forecasted volume (n=77). Within firearm conversations, “grief” issue tags showed a significant increase in proportion in the week following the shooting, while “isolation/loneliness,” “relationships,” and “suicide” issue tags showed a significant decrease in proportions the week following the shooting.

**Conclusions:**

The results suggest that the Uvalde school shooting may have contributed to an increase in demand for crisis services, above what would be expected given historical trends. Additionally, we found that these firearm-related crises conversations immediately post event are more likely to be related to grief and less likely to be related to suicide, loneliness, and relationships. Our findings provide some of the first data showing the real-time repercussions for the broader population exposed to school shooting events. This work adds to a growing evidence base documenting and measuring the rippling effects of mass shootings outside of those directly impacted.

## Introduction

Mass shootings result in widespread psychological distress and trauma symptoms for survivors and members of the impacted community [1]. Research demonstrates that following a mass shooting, higher rates of psychological symptoms (eg, depressive symptoms, increased feelings of fear, and decreased perceptions of safety) are found in the immediately affected community [2]. While there is emerging evidence suggesting that mass shootings may have negative mental health consequences that reach beyond local communities to broader populations through indirect exposure (eg, conversations, media exposure) [2], information about immediate effects of mass shootings on broader public well-being is nearly absent in the literature. One study showed that teachers who had increased exposure to school shooting–related media had increased experiences of secondary trauma [3]. Advancing understanding of indirect exposure and resulting mental health–related issues will inform future public health response.

There are several challenges associated with studying the effects of indirect exposure to mass shootings using traditional survey methods. Mass shooting events are unpredictable low-base rate events. Scheduled data collection of longitudinal or repeated cross-sectional studies may not align with when mass shootings occur, leading to excessively inefficient and costly survey operations. Furthermore, prolonged periods between exposure and data collection may lead to recall bias [4], reducing the quality of responses and the ability to make strong inferences. Recently, alternative study designs have been used to address these methodological challenges. For example, studies have analyzed the content of social media posts before and after mass shooting events to show increases in expression of emotions in the general population [5,6].

Data from crisis lines provide a unique opportunity to examine real-time data on the widespread psychological effects of mass shootings. Crisis Text Line (CTL) is a not-for-profit company that provides 24/7 confidential crisis counseling via SMS text messaging and WhatsApp. The majority of texters served by CTL in the United States are younger than 25 years (76% aged <25 years) [7]. CTL provides rich data specifically focused on mental health–related crises in the moments that they occur. Previous studies have used time series research designs with deidentified CTL data to analyze changes in the number and types of crises due to exposure to *13 Reasons Why* on Netflix [8], celebrity suicide deaths [9], and Hurricane Florence [10]. These prior investigations collectively demonstrate that poignant events that have widespread coverage on various media outlets (including social media) have measurable impact on the larger corpus of individuals texting CTL.

In this study, we investigated CTL’s anonymized conversations related to firearm violence, including mass shootings, suicide, and other firearm-related issues. To our knowledge, no existing study has examined firearm-related crises in CTL conversations or other crisis lines. We examined changes in the volume and composition of CTL firearm-related texts before and after the Robb Elementary School mass school shooting on May 24, 2022, in Uvalde, Texas. Based on previous investigations with deidentified CTL data and national events [8-10], we hypothesized that conversations mentioning firearms would increase in the days following the mass shooting. Because work has not examined the broader effects of indirect exposure to mass shootings on mental health, we did not have a specific hypothesis about how social and mental health–related stressors may change before and after a mass shooting event. The objective of this study was to provide direct evidence of the effects of mass shootings on the broader public’s mental health.

## Methods

### Overview

Anonymized and deidentified full crisis conversation transcripts and postconversation survey data completed by volunteer crisis counselors (vCCs) was obtained from CTL for the dates surrounding the Robb Elementary School shooting (March 1 to May 30, 2022). We filtered the anonymized and deidentified individual CTL conversations by 123 firearm-related keywords (eg, “gun,” “shooting”) to create a corpus of firearm-related crisis conversations. The keyword list started with common terms for firearms and was expanded using a word embedding similarity search [11] to discover firearm terms in the conversation text that were not present in the initial list (eg, “gsw,” “9 mm”), expanding the number of keyword terms from 23 to 37. Word embeddings were generated using the Word2Vec algorithm [12] trained on all CTL conversations from September 2018 to August 2021 (N=2,539,460 conversations), which is the date range of focus for additional ongoing projects with the CTL data. Keywords were expanded to 123 words by examining conversations where vCCs’ postconversation survey notes had a firearm keyword listed as the potential means of suicide but that were not already identified based on the conversation text using our existing keyword list. A full list of keywords is available in Multimedia Appendix 1.

A quasi-experimental event study design [13] was used to compare the actual firearm conversation volume post event to forecasted firearm conversation volume under the counterfactual scenario that a shooting had not occurred. Expected volumes were estimated using a seasonal autoregressive integrated moving average time series model with an order of (0, 1, 1) and seasonal order of (1, 1, 1, 7) on daily counts for a pre-event period (March 1 to May 23, 2022) to forecast the firearm conversation volume post event (May 4 to May 30, 2022). Other mass shooting events in the pre-event period were incorporated into the model using an exogenous dummy variable (1=mass shooting, 0=no shooting). Other mass shooting events were identified using the Gun Violence Archive [14] and the US Congress definition of mass public shooting [15]. A start date of March 1, 2022, was chosen to focus on recent months, though a start date of January 1, 2022, was also tested with similar results. An end date of May 30, 2022, was chosen to exclude the June 1, 2022, mass shooting in Tulsa, OK.

Lastly, we compared proportions of conversation issue tags (characteristics of the text conversation coded by CTL vCCs during/after the exchange) 4 days before (n=251) and after (n=417) the shooting to assess changes in conversation characteristics. The duration of the time before and after the event was chosen empirically to represent days in which the actual volume of firearm-related conversations exceeded the expected. Chi-square tests were used to test the hypothesis that issue tag proportions differed in the pre- and postevent periods.

### Data Management

Data from CTL was accessed on a secure remote server hosted by CTL. All data used in this study will be deleted upon completion of the broader Centers for Disease Control and Prevention–funded research project.

### Ethical Considerations

This project was reviewed by the RTI Office of Research Protection Institutional Review Board (IRB; STUDY00021510) and deemed to fall under the category of Not Human Subjects Research (public health practices and surveillance activities). Data used within this study are collected by existing infrastructure at CTL as part of the crisis services they provide. All data gathered from texters including text conversations and postconversation survey data provided by CTL are anonymized and deidentified before transfer to the research team for analysis. Texters agree to the use of their anonymized information for research purposes as part of the CTL’s terms of service automatically shared before being connected with a vCC [16]. Anonymized texter information is shared for public good in aggregate at CrisisTrends.org and with select researchers following a rigorous screening process and IRB approval, and texters can request that their conversations be removed from CTL’s systems at any time following their conversations.

## Results

**Figure 1:**
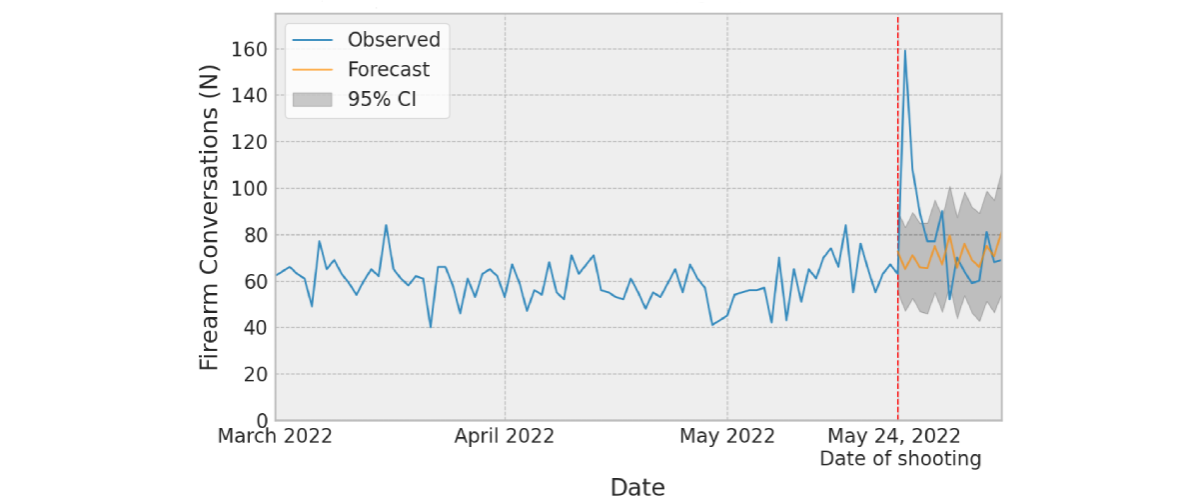
Count of Crisis Text Line firearm-related conversations following the Robb Elementary School shooting.

We observed a significant increase in the number of conversations mentioning firearms following the shooting at Robb Elementary School (Figure 1). The largest spike in conversations mentioning firearms occurred the day after the shooting (n=159), May 25, 2022, with volume reverting to within the 95% CI of forecasted volume 4 days after the shooting on May 28 (n=77).

Table 1 reports the frequency and proportions of issue tags for conversations in the immediate 4-day pre- and postevent period. For firearm conversations during the postevent period, the count of all issue tags increased, except for the “COVID-19” issue tag. To control for increases in tags post event, we examined the percentage of total firearm conversations in the pre- and postevent period that each unique tag was used. Comparing pre-event to postevent, the “grief” issue tag increased significantly in the days following the shooting (*d*=0.06; *P*=.03), while the proportions for the “isolation/loneliness” (*d*=–0.07; *P*=.04), “relationships” (*d*=–0.11; *P*<.001), and “suicide” (*d*=–0.14; *P*<.001) issue tags decreased in the days following the shooting.

**Table 1:** Issue tag proportions for conversations prior and in the week following the shooting sorted by pre-post difference in proportions.

Issue tag	Count before, n	Count after, n	Pre-event proportion (n=251)	Postevent proportion (n=417)	Difference	*P* value
Grief	19	55	0.08	0.13	0.06	*.03* ^a^
Anxiety	79	154	0.31	0.37	0.05	.15
Abuse (emotional)	8	17	0.03	0.04	0.01	.56
Gender/sexual identity	3	7	0.01	0.02	0.00	.62
Racism	4	8	0.02	0.02	0.00	.76
Depression/sadness	6	11	0.37	0.37	0.00	.99
Eating disorder/body image	92	154	0.02	0.02	0.00	.99
Abuse (physical)	6	8	0.02	0.02	0.00	.68
COVID-19	3	2	0.01	0.00	–0.01	.03
Abuse (unspecified)	7	8	0.03	0.02	–0.01	.46
Abuse (sexual)	10	12	0.04	0.03	–0.01	.44
Bullying	10	11	0.05	0.03	–0.01	.36
Substance use	12	14	0.04	0.02	–0.02	.24
Self-harm	27	34	0.11	0.08	–0.03	.26
Isolation/loneliness	69	87	0.27	0.21	–0.07	*.04*
Relationships	95	112	0.39	0.26	–0.13	*<.001*
Suicide	118	141	0.47	0.34	–0.13	*<.001*

^a^Italics indicate values that are statistically significant.

## Discussion

The objective of this investigation was to examine the effect of a mass shooting event on the broader public’s mental health. To do so, we examined the immediate effects of a widely publicized mass shooting in the United States on volume of mental health crisis text conversations that mentioned firearms. We also examined the psychosocial context of those text conversations by investigating changes in issue tags associated with firearm conversations as coded by the vCCs. Consistent with our hypothesis, we found that firearm-related crisis conversations following the Uvalde school shooting significantly increased. In addition to this main finding, we observed an increase in the proportion of grief-related conversations that mentioned firearms and a decrease in suicide-related conversations that mentioned firearms. We also observed a decrease in firearm conversations associated with relationships or feelings of isolation/loneliness following the Uvalde school shooting. Overall, we saw evidence that a widely publicized mass shooting influenced the broader public’s mental health beyond the specific location of the mass shooting event.

Our innovative methodological approach of analyzing real-time mental health crises through CTL conversations allowed us to address a critical gap in knowledge of the psychological effects of mass shootings on remote populations of individuals who are not directly exposed to the mass shooting event. Only a couple of prior studies have investigated the effects of mass shooting events on youth populations beyond the communities directly involved in these events, and these studies have mainly focused on youth expressions of emotion such as fear. However, our study findings may have the potential to have a significant impact on mental health crises (beyond general expressions of negative emotions) among the public. Interestingly, the increase in crisis conversation volume existed even when accounting for other mass shootings in the study period. It is possible that the Uvalde shooting has a particularly strong impact on users of CTL because school shootings (relative to other forms of mass shootings) appear to significantly affect youth mental health [17], and the CTL population is largely composed of youth [7]. Of course, other confounding factors such as the amount of media coverage or social media mentions may also be influential.

The increase in proportion of grief-related conversations, along with the decrease in suicide-related conversations, further suggest that firearm crisis conversations post event may be focused more on loss rather than suicide as is typically more common in firearm-related mental health crisis conversations. These findings are consistent with prior research showing that grief is a common reaction immediately following school shootings [18]. We also observed a decrease in firearm conversations associated with isolation and relationship issues. Although the data does not illustrate why this shift occurred, we speculate that an increase in social solidarity after the mass shooting event leads to fewer crises due to isolation and relationship factors. This possibility is consistent with prior studies suggesting that community members often reach out to provide more emotional support to one another after traumatic events [19]. Together, our results demonstrate that firearm-related mental health crisis conversations immediately following a mass shooting differ from typical firearm conversations in that they are more likely to be associated with grief issues and less likely to be associated with suicide, isolation, and relationship issues.

Several limitations of the study should be noted. First, we assume that the firearm conversations represent users that were not directly impacted by the Uvalde shooting (survivors, direct family members, etc) due to the relatively smaller number of people expected to incur direct versus indirect effects. Future studies should use geographic indicators to increase the chance of examining those directly related to a mass shooting event. Second, our study design does not address why the text volume associated with the Uvalde school shooting is greater than other mass shootings observed in the study period. Future research studying the magnitude of indirect impacts of mass shootings across different types of events could greatly enhance the literature, for example, the impact of school shootings compared to nonschool shootings. Additionally, this study does not attempt to quantify the impact of increased awareness of or referrals to CTL due to the Uvalde shooting. Lastly, issue tags were designated by the vCCs and thus relatively subjective. Future work could study the reliability of vCCs’ tagging to help better understand how mass shootings may be related to increases in firearm-related mental health crises. Overall, our findings support two main conclusions. First, the Uvalde, TX mass school shooting increased firearm-related text conversations at a national mental health crisis line even after factoring in the effects of other mass shootings that occurred proximally in time. Our findings provide some of the first data showing the real-time repercussions for the broader population exposed to school shooting events. Public health officials must recognize that the reverberations of school shootings on mental health extend beyond just the immediate community where the event takes place. Second, these firearm-related crises conversations may be most frequently related to grief. Thus, findings suggest that public health interventions that target feelings of grief may have the potential to reduce mental health crises immediately following a school mass shooting. Overall, the findings lay the foundation for future research on effective public mental health interventions that may be implemented after school mass shootings.

## Appendix

Multimedia Appendix 1 Firearm keywords used in conversation identification.

## Abbreviations

CTL: Crisis Text Line
vCC: volunteer crisis counselor

## Data Availability

Data from Crisis Text Line used in this research is not available publicly due to concerns of privacy and anonymity of texters who use Crisis Text Line’s service. Aggregated anonymized data from more than 7 million conversations with people in crisis supported by Crisis Text Line is available at CrisisTrends.org.
