# Supplementary material for: The Use of Crisis Services Following the Mass School Shooting in Uvalde, Texas: Quasi-Experimental Event Study
Source: JMIR Public Health Surveill. 2023 Feb 8;9:e42811. doi: 10.2196/42811 (PMC9947763; doi:10.2196/42811)
Supplement: Multimedia Appendix 1 [file publichealth_v9i1e42811_app1.docx]

**Multimedia Appendix 1.** Firearm keywords used in conversation identification.

1. handgun
2. shotgun
3. gun
4. revolver
5. firearm
6. handguns
7. shotguns
8. guns
9. firearms
10. pistols
11. rifles
12. bb gun
13. pellet gun
14. crossbow
15. bb guns
16. glock
17. ar15
18. ar-15
19. ak47
20. 357
21. .308
22. .357
23. 12ga
24. .380
25. 9 mm
26. .22
27. .45
28. .38
29. .40
30. chambered
31. holster
32. misfired
33. bullet
34. pellet
35. gunsafe
36. bullets
37. buckshot
38. gsw
39. gunshot
40. 9mm
41. weapon
42. shooting me
43. shooting us
44. shooting you
45. shooting him
46. shooting her
47. shooting it
48. shooting them
49. shot me
50. shot us
51. shot you
52. shot him
53. shot her
54. shot it
55. shot them
56. shoot me
57. shoot us
58. shoot you
59. shoot him
60. shoot her
61. shoot it
62. shoot them
63. i shot
64. we shot
65. he shot
66. she shot
67. it shot
68. they shot
69. shooting myself
70. shooting my self
71. shooting ourselves
72. shooting our selves
73. shooting yourself
74. shooting your self
75. shooting yourselves
76. shooting your selves
77. shooting himself
78. shooting him self
79. shooting herself
80. shooting her self
81. shooting itself
82. shooting it self
83. shooting themself
84. shooting them self
85. shooting themselves
86. shooting them selves
87. shot myself
88. shot my self
89. shot ourselves
90. shot our selves
91. shot yourself
92. shot your self
93. shot yourselves
94. shot your selves
95. shot himself
96. shot him self
97. shot herself
98. shot her self
99. shot itself
100. shot it self
101. shot themself
102. shot them self
103. shot themselves
104. shot them selves
105. shoot myself
106. shoot my self
107. shoot ourselves
108. shoot our selves
109. shoot yourself
110. shoot your self
111. shoot yourselves
112. shoot your selves
113. shoot himself
114. shoot him self
115. shoot herself
116. shoot her self
117. shoot itself
118. shoot it self
119. shoot themself
120. shoot them self
121. shoot themselves
122. shoot them selves
123. shooting
